# Interleukin-3 is associated with sTREM2 and mediates the correlation between amyloid-β and tau pathology in Alzheimer’s disease

**DOI:** 10.1186/s12974-022-02679-5

**Published:** 2022-12-29

**Authors:** Zhi-Bo Wang, Ya-Hui Ma, Yan Sun, Lan Tan, Hui-Fu Wang, Jin-Tai Yu, for the Alzheimer’s Disease Neuroimaging Initiative

**Affiliations:** 1 Department of Neurology, Qingdao Municipal Hospital, Qingdao University, No. 5 Donghai Middle Road, Qingdao, China; 2 Institute of Science and Technology for Brain-Inspired Intelligence, Fudan University, Shanghai, China; 3 State Key Laboratory of Medical Neurobiology and MOE Frontiers Center for Brain Science, Department of Neurology and Institute of Neurology, Huashan Hospital, Shanghai Medical College, Fudan University, Shanghai, China

**Keywords:** Alzheimer’s disease, Amyloid-β, Interleukin-3, Tau, Microglia, Astrocyte

## Abstract

**Background:**

Dysfunction of glial cell communication is involved in Alzheimer’s disease (AD) pathogenesis, and the recent study reported that astrocytic secreted interleukin-3 (IL-3) participated in astrocyte–microglia crosstalk and restricted AD pathology in mice, but the effect of IL-3 on the pathological progression of AD in human is still unclear.

**Methods:**

A total of 311 participants with cerebrospinal fluid (CSF) IL-3, soluble triggering receptor expressed on myeloid cells 2 (sTREM2), and AD biomarkers were included from the Alzheimer’s disease Neuroimaging Initiative (ADNI). We assessed the associations of IL-3 with sTREM2 and AD biomarkers at baseline, and with cognitive change in longitudinal study. The mediation models were used to explore the potential mechanism of how IL-3 affects AD pathology.

**Results:**

We found that CSF IL-3 was significantly associated with CSF sTREM2 and CSF AD core biomarkers (Aβ42, p-tau, and t-tau) at baseline, and was also markedly related to cognitive decline in longitudinal analysis. Moreover, mediation analysis revealed that CSF IL-3 modulated the level of CSF sTREM2 and contributed to tau pathology (as measured by CSF p-tau/t-tau) and subsequent cognitive decline. In addition, Aβ pathology (as measured by CSF Aβ42) affected the development of tau pathology partly by modifying the levels of CSF IL-3 and CSF sTREM2. Furthermore, the effect of Aβ pathology on cognitive decline was partially mediated by the pathway from CSF IL-3 and CSF sTREM2 to tau pathology.

**Conclusions:**

Our findings provide evidence to suggest that IL-3 is linked to sTREM2 and mediates the correlation between Aβ pathology to tau pathology. It indicates that IL-3 may be a major factor in the spreading from Aβ pathology to tau pathology to cognitive impairment.

**Supplementary Information:**

The online version contains supplementary material available at 10.1186/s12974-022-02679-5.

## Background

Alzheimer’s disease (AD) is the most common type of dementia, characterized by two specific neuropathological profiles: extracellular amyloid-β (Aβ) deposition and intraneuronal neurofibrillary tangles (NFTs) consisting of aggregated hyperphosphorylated tau protein [1]. Glial cells were believed primarily to contribute to maintaining central nervous system (CNS) function that plays a crucial role in the development of AD dementia [2]. Astrocyte and microglia, as two common types of glial cells, serve as the key regulator promptly responding to inflammatory signals that orchestrate inflammatory responses and helps restore CNS homeostasis [3–5]. However, excessive glial cell activation may be a direct pathway that causes Aβ deposition, tau pathology, and neuronal damage in AD [1, 6]. Crosstalk within astrocytes and microglia may contribute to maintaining their normal function both in healthy physiology and pathology, especially when responding to insult or injury [3, 7]. Astrocytes can produce molecules to regulate microglial phenotypes and functions ranging from motility to phagocytosis, and microglia also can determine the functions of reactive astrocytes, ranging from neuroprotective to neurotoxic [8–10]. The astrocyte–microglia communication is fundamental to neuronal functions and dysfunctions. However, the role of astrocyte–microglia communication in the pathogenesis of AD is poorly understood.

Interleukin-3 (IL-3), a multifunctional cytokine involved in astrocyte–microglia communication, is believed to involve in inflammatory and autoimmune diseases [11]. Cerebrospinal fluid (CSF) IL-3 secreted from astrocytes has a distinctive function on the proliferation and programming of microglial cells [12, 13]. Beyond the mediated role of IL-3 on normal immune homeostasis, recent studies have applied IL-3 and other signaling proteins in blood to predict the occurrence of AD [14, 15] as well as the primary pathological traits of AD (such as Aβ and tau-related pathology) [16–18]. Preclinical work using mice models has shown that IL-3 can prevent neuronal death induced by amyloid pathology [19] and can attenuate tau-related pathology [20]. In addition, animal model evidence indicated that the beneficial effect of IL-3 on the brain may act as a triggering receptor expressed on myeloid cells 2 (TREM2)-dependent manner to allow microglia to counteract the detrimental Aβ plaque deposition, then promoting memory function [12]. Autopsy evidence also indicated that IL-3 signaling was associated with microglial activation and AD pathology [12]. However, no population-based studies have investigated this question using CSF biomarkers, which would allow in vivo characterization of AD pathology in larger and more generalized samples in the early course of the disease. Thus, in this study, we aimed to explore the role of IL-3 and microglia on AD pathology in a larger population-based cohort of older adults.

To systematically determine the complex roles of IL-3 on AD pathogenesis and its specific interactions with microglial activity in the participants who had preclinical or clinical AD, we aimed to ascertain the interrelationships between CSF IL-3, microglial activation markers (as reflected by soluble triggering receptor expressed on myeloid cells 2 [sTREM2]), AD pathology, and cognitive change, and to explore effects of CSF IL-3 and CSF sTREM2 on AD pathology and cognitive change using a mediation model. We hypothesized that CSF IL-3 was associated with CSF sTREM2 and AD pathology as well as cognitive change and that association of CSF IL-3 and CSF sTREM2, as reflected in astrocyte–microglia communication, may be a key part of the progression of AD pathology, and even with subsequent cognitive impairment.

## Methods

### Study participants

This study included participants from the Alzheimer’s Disease Neuroimaging Initiative (ADNI) database. The ADNI project, a multicenter longitudinal study, aims to combine clinical, imaging, genetic, and biochemical biomarkers to develop and validate the measures of early diagnosis of late-onset AD. The detailed inclusion criteria in ADNI can be found at www.adni-info.org. ADNI was approved by the institutional review boards of all participating institutions and all participants provided written informed consent.

**Fig. 1:**
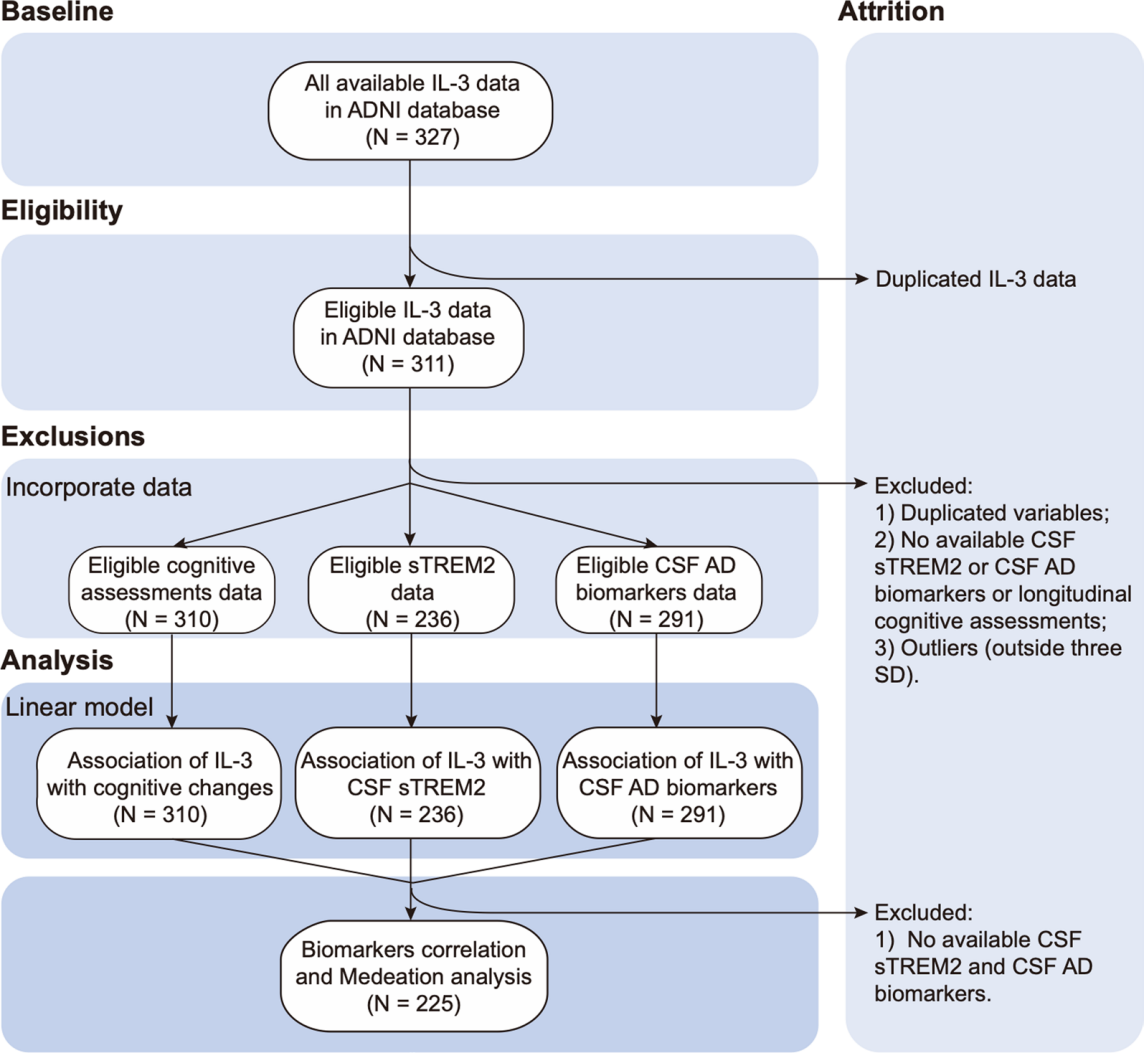
Participant flowchart. Flowchart depicting the step-by-step selection process of the Alzheimer’s Disease Neuroimaging Initiative subjects included for data analyses

A total of 327 received measurements of CSF IL-3 in the ADNI database. Among them, participants who completed the detection of CSF sTREM2, AD biomarkers (Aβ42, total tau[t-tau], and phosphorylated tau[p-tau]), and followed up cognitive measures at least 1 years (baseline plus two follow-up visits) were included in this study (see Fig. 1 for detailed sample selection). In addition, to test whether IL-3 levels were influenced by comorbidities, we performed sensitivity analysis in populations by excluding those diagnosed with sepsis, rheumatoid arthritis, acute lymphoblastic leukemia, asthma, multiple sclerosis, depression, and schizophrenia in medical history and medical record (total 44 participants were excluded), because previous studies have shown a strong relationship between these diseases and IL-3 [21–27]. In the present study, *APOE4* status were grouped as carrying at least one *APOE4* allele or no one *APOE4* allele.

### Measurements of CSF IL-3, sTREM2, and AD biomarkers

The CSF level of AD core biomarkers (Aβ42, p-tau, and t-tau) was measured using the electrochemiluminescence immunoassays Elecsys immunoassays on a cobas 601 instrument. CSF sTREM2 levels were measured by the MSD platform-based assay, and previous publication reported and validated this detailed method [28]. CSF IL3 levels were measured by Rules Based Medicine (RBM, Austin, TX) using Luminex xMAP technology in ADNI database. Raw data for IL-3 were normalized using log transformed in ADNI database. Detailed methods of analyte measurements can be found online in the ADNI LONI Image & Data Archive (http://adni.loni.usc.edu).

### Cognitive assessments

ADNI composite memory score (ADNI-MEM) and ADNI composite executive function score (ADNI-EF) were applied to reflect memory function and executive function, respectively [29]. Alzheimer Disease Assessment Scale (ADAS) 11, ADAS 13 [30], MMSE [31], and CDR scores were applied to reflect global cognition. Notably, higher levels of ADAS 11 scores, ADAS 13 scores, and CDR scores and lower levels of ADNI-MEM, ADNI-EF, and MMSE scores indicate worse cognitive performance.

### Statistical analysis

The normality of distribution for each biomarker was assessed by Kolmogorov–Smirnov test, and those variables that did not follow a normal distribution were normalized by the Box–Cox transformations. Outliers (outside three SD) were excluded from this study. Based on pre-established CSF AD biomarker cutoffs [32], participants in this study were defined as CSF A ± (CSF Aβ42 < 976.6 pg/ml or ≥ 976.6 pg/ml), T ± (p-tau > 21.8 pg/ml or ≤ 21.8 pg/ml), and N ± (t-tau > 245 pg/ml or ≤ 245 pg/ml). We used previously reported methods that merged the aggregated tau (*T*) and neurodegeneration (N) groups and then, subjects were categorized into different A/T/N categories: Stage 0 (A−TN−), Stage 1 (A+TN−), Stage 2 (A+TN+), Suspected Non-AD Pathology (SNAP) (A−TN+) [33]. Baseline characteristics were compared using one-way analysis of covariance (ANOCVA) for continuous variables and *χ*^2^ tests for categorical variables. The associations between IL-3 with sTREM2 and AD biomarkers were explored utilizing Spearman partial correlation and multiple linear regression (MLR), with adjustment for age, sex, education, and *APOE4* status. Next, we tested whether baseline IL-3 levels were associated with longitudinal cognitive change using a linear mixed model, controlling follow-up time, random slope, intercept, age, sex, education, and *APOE4* status.

Finally, five mediation models using structural equation model (SEM) were conducted in this study. First, we tested whether the association between Aβ42 and sTREM2 was mediated by IL-3. The second mediation model was used to explore whether the association between IL-3 and p-tau/t-tau was mediated by sTREM2. The serial mediation model was to explore whether associations of Aβ42 with p-tau/t-tau were mediated by IL-3 and sTREM2. We calculated the subject-specific slopes as the annual change rates of cognitive change in a linear model cognitive outcome by time and the fourth mediation model was tested whether association of IL-3 and cognitive change was mediated by sTREM2 and p-tau/t-tau. The last mediation model was used to explore whether association of Aβ42 with cognitive change was mediated by IL-3, sTREM2, and p-tau/t-tau. These mediation models were both adjusting age, sex, education, and *APOE4* status.

A two-sided *p* value < 0.05 was considered statistically significant. The “lmer”, “lme4”, “corrplot”, “lavaan”, “ggplot2”, and “car” packages in R version 4.1.0 software were used to perform the all above analyses.

## Results

### Participant characteristics

Demographic, clinical, and biomarker characteristics of the samples are reported in Table 1. We included 255 participants in Table 1 including 43 stage 0, 34 stage 1, 118 stage 2, and 30 SNAP. The mean age of participants in this study was 75.3 (± 6.6) years, with 39% female. There was a significant difference between four different A/T/N groups including *APOE4* allele, MMSE scores, and AD diagnosis as well as CSF IL-3, CSF sTREM2, CSF Aβ42, CSF t-tau, and CSF p-tau, but not age, sex, and education (Table 1).

**Table 1:** Participant characteristics at baseline by biomarker-defined groups in the ADNI database

Characteristic	Stage 0	Stage 1	Stage 2	SNAP	*P* value
*N*	43	34	118	30	–
Age, mean (SD), y	75.28 (5.4)	75.25 (5.4)	74.81 (7.2)	77.11 (7.3)	0.413
Female, *n* (%)	15 (34.9)	9 (26.5)	53 (44.9)	11 (36.7)	0.226
Education, mean (SD), y	15.58 (3.0)	15.50 (3.7)	15.73 (3.1)	15.87 (2.8)	0.961
*APOE ε4* carriers, *n* (%)	4 (9.3)	16 (47.1)	85 (72.0)	6 (20.0)	< 0.001
MMSE score, mean (SD)	28.53 (1.3)	26.38 (2.5)	25.93 (2.7)	28.13 (1.9)	< 0.001
AD diagnosis, *n* (%)	1 (2.3)	6 (17.6)	39 (33.1)	3 (10.0)	< 0.001
CSF IL-3, mean (SD)	− 2.12 (0.3)	− 2.37 (0.3)	− 2.21 (0.3)	− 1.97 (0.3)	< 0.001
CSF biomarkers, mean (SD), pg/ml					
CSF sTREM2	4451.21 (2129.9)	3043.37 (1284.5)	4547.32 (2000.8)	5446.16 (1895.3)	< 0.001
CSF Aβ42	1445.07 (256.5)	632.19 (199.7)	609.02 (167.5)	1549.27 (428.8)	< 0.001
CSF p-tau	16.89 (2.8)	16.38 (3.7)	35.95 (10.0)	29.00 (10.7)	< 0.001
CSF t-tau	191.95 (31.4)	178.69 (34.7)	356.87 (91.6)	314.17 (86.7)	< 0.001

*Aβ* β-amyloid, *AD* Alzheimer’s disease, *CSF* cerebrospinal fluid, *IL-3* interleukin-3, *MMSE* Mini-Mental State Examination, *p-tau* phosphorylated tau, *t-tau* total tau

*P* values were computed with the one-way analysis of covariance test for age, education, MMSE score, CSF IL-3, CSF sTREM2, CSF Aβ42, CSF t-tau, CSF p-tau; with the *χ*^2^ test for sex and *APOE* status

### Association of IL-3 with sTREM2 and AD biomarkers

**Fig. 2:**
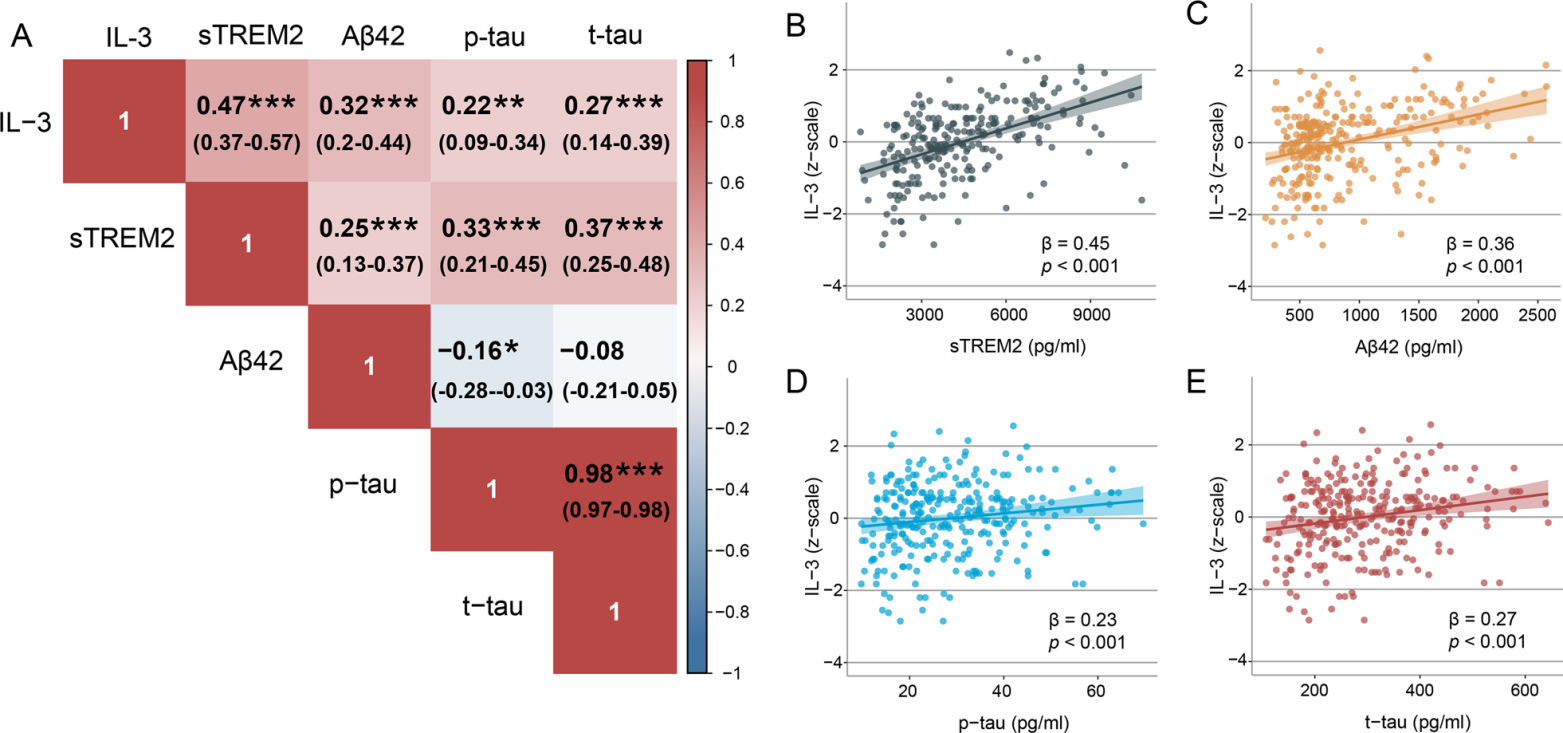
Biomarker correlations. **A** The left panel shows biomarkers correlations. Red indicates positive correlation, and blue indicates negative correlation. The Spearman partial correlation coefficients (*r*) and confidence interval are shown in each square after controlled age, sex, education, and *APOE4* status. *P < 0.05, **P < 0.01 and ***P < 0.001. **B**–**E** Scatter plots show the associations of CSF IL-3 with CSF sTREM2, CSF Aβ42, CSF p-tau, and CSF t-tau. The normalized regression coefficients (*β*) and *P* values shown in scatter plots were derived from multiple linear regression. Linear model fits are indicated together with 95% confidence intervals. These models were adjusting age, sex, education, and *APOE4* status

We first sought to test the association between CSF IL-3 with CSF sTREM2 and CSF AD biomarkers. CSF IL-3 were positively correlated with CSF sTREM2 (*β* = 0.45, *p* < 0.001) and showed a strong correlative trend with CSF Aβ42 and CSF p-tau as well as CSF t-tau (Fig. 2B–E). In addition, the association results with other CSF biomarkers from MLR model are presented in Fig. 2A.

### Association of CSF IL-3 with cognitive changes

**Fig. 3:**
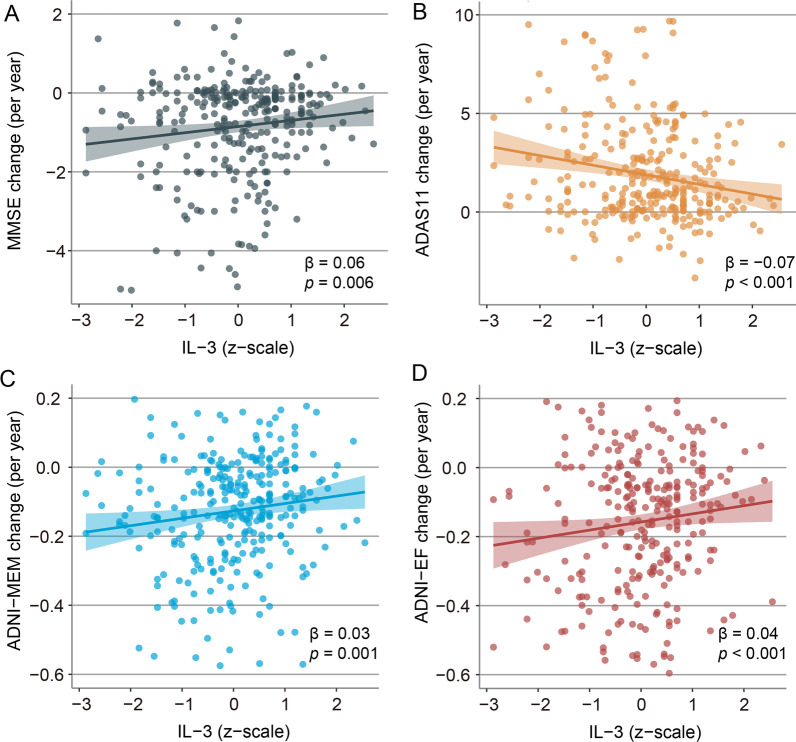
Effect of baseline CSF IL-3 levels on changes in cognition. **A**–**D** Scatterplots display the relationships between baseline CSF IL-3 and annual change rates of MMSE, ADAS 11, ADNI-MEM, and ADNI-EF. Linear model fits are indicated together with 95% confidence intervals. The normalized regression coefficients (*β*) and *P* values shown in scatter plots were derived from the interaction term of CSF IL-3 × time in the linear mixed regression model, controlled for CSF IL-3, follow-up years, age, sex, education, and *APOE4* status

We next studied the association between baseline CSF IL-3 with subsequent cognitive changes. For the global cognitive scores, the interaction of CSF IL-3 × time was significantly associated with longitudinal MMSE scores (Fig. 3A), ADAS11 scores (Fig. 3B), CDR scores (*β* = − 0.053, *p* = 0.006), and ADAS13 scores (*β* = − 0.053, *p* < 0.001) (Additional file 1: Table S1). Specifically, for memory and executive function, the interaction of CSF IL-3 × time was also significant both in ADNI-MEM scores (Fig. 3C) and ADNI-EF scores (Fig. 3D) (Additional file 1: Table S1).

### Association of CSF IL-3 with tau pathology mediated by CSF sTREM2

To explore the influences of IL-3 and sTREM2 on AD pathology, two mediation pathway analyses were used including: (1) CSF Aβ42 → CSF IL-3 → CSF sTREM2; (2) CSF IL-3 → CSF sTREM2 → CSF p-tau/t-tau. In the first mediation model, Aβ42 had a positive association with IL-3 (*β* = 0.314, *p* < 0.001) and sTREM2 (*β* = 0.22, *p* < 0.001). The indirect effect of Aβ42 on sTREM2 via IL-3 was significant (*β* = 0.134, *p* < 0.001) (Additional file 1: Fig. S1A). In the second mediation model, IL-3 also had a positive association with sTREM2 (*β* = 0.453, *p* < 0.001) and p-tau (*β* = 0.231, *p* < 0.001) as well as t-tau (*β* = 0.274, *p* < 0.001) (Additional file 1: Fig. S1B). The results of second mediation model showed that sTREM2 mediates associations between IL-3 and p-tau (*β* = 0.122, *p* < 0.001) as well as t-tau (*β* = 0.127, *p* < 0.001) (Additional file 1: Fig. S1B, C).

### CSF IL-3 and sTREM2 are key mediators in the association between amyloid pathology and tau pathology

**Fig. 4:**
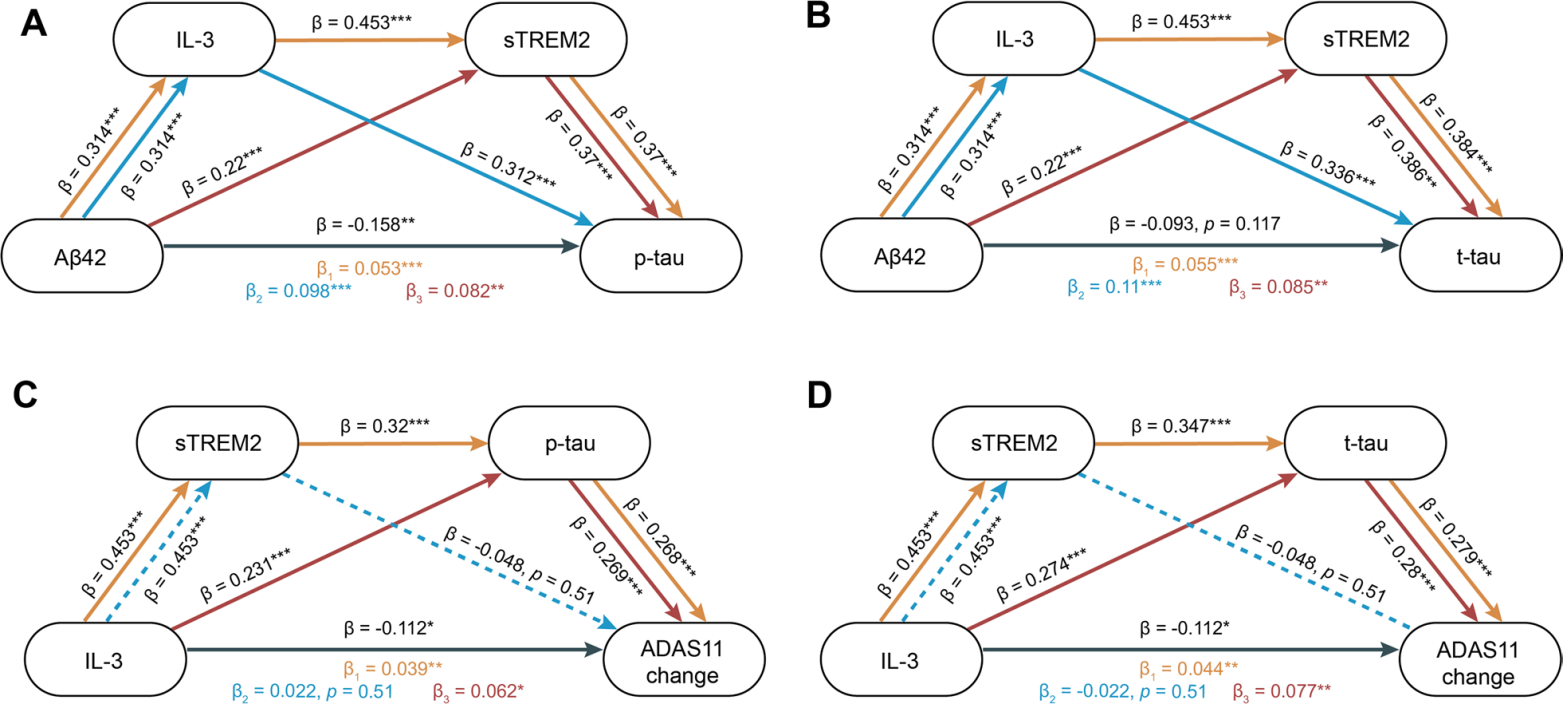
Mediation analysis. **A**, **B** Three mediation pathways were conducted between Aβ42 and p-tau/t-tau: (1) Aβ42 → IL-3 → sTREM2 → p-tau/t-tau; (2) Aβ42 → IL-3 → p-tau/t-tau; (3) Aβ42 → sTREM2 → p-tau/t-tau. **C**, **D** Three mediation pathways were assessed between IL-3 and ADAS11 annual change: (1) IL-3 → sTREM2 → p-tau/t-tau → ADAS11 change; (2) IL-3 → sTREM2 → ADAS11 change; (3) IL-3 → p-tau/t-tau → ADAS11 change. These three pathways are presented using yellow, blue, and red lines. All mediation paths are adjusted by covariates (age, sex, education, and *APOE4* status). The *β* coefficients in each path and *P*-values for mediation effects were calculated by a bootstrap test with 10,000 resampling iterations. The dotted line indicates that the indirect effect is not significant (*P* ≥ 0.05), and the solid line indicates that the indirect effect is significant (*P* < 0.05). *P < 0.05, **P < 0.01 and ***P < 0.001

We further tested whether IL-3 and sTREM2 contributed to the effect of Aβ42 on subsequent tau pathology. Therefore, the third mediation model including three mediation pathway analyses were used: (1) CSF Aβ42 → CSF IL-3 → CSF p-tau/t-tau; (2) CSF Aβ42 → CSF sTREM2 → CSF p-tau/t-tau; (3) CSF Aβ42 → CSF IL-3 → CSF sTREM2 → CSF p-tau/t-tau. In the first mediation model, Aβ42 was positively associated with IL-3 and was negatively associated with p-tau/t-tau, and IL-3 was positively associated with p-tau/t-tau. The indirect effect of Aβ42 on p-tau/t-tau via IL-3 was significant (Fig. 4A, B). In the second mediation model, Aβ42 had a positive association with sTREM2 and sTREM2 also had a positive association with p-tau/t-tau. The results of second mediation model showed that sTREM2 mediates associations between Aβ42 and p-tau/t-tau (Fig. 4A, B). The third model revealed a significant mediation effect of IL-3 and/or sTREM2 on the association between Aβ42 and p-tau/t-tau (Fig. 4A, B).

The fourth mediation model including three pathways determined whether CSF sTREM2 and CSF p-tau/t-tau contributed to the association between CSF IL-3 and cognitive change: (1) CSF IL-3 → CSF sTREM2 → CSF p-tau/t-tau → ADAS11 change; (2) CSF IL-3 → CSF sTREM2 → ADAS11 change; (3) CSF IL-3 → CSF p-tau/t-tau → ADAS11 change. The significant associations were observed between p-tau/t-tau with ADAS11 change. However, sTREM2 did not reach a significant association with ADAS11 change (Fig. 4C, D). The serial mediation pathway revealed that the effect of IL-3 on ADAS11 change via sTREM2 and p-tau/t-tau was significant (Fig. 4C, D). The results of third pathway showed that p-tau/t-tau were also separately significant mediators for this association, but not sTREM2 (Fig. 4C, D). As a sensitivity analysis, five other cognitive assessments were used to conduct mediation analysis, including MMSE, ADNI-MEM, ADAS 13, ADNI-EF, and CDR, and the similar mediation results remained (see Additional file 1: Fig. S2).

**Fig. 5:**
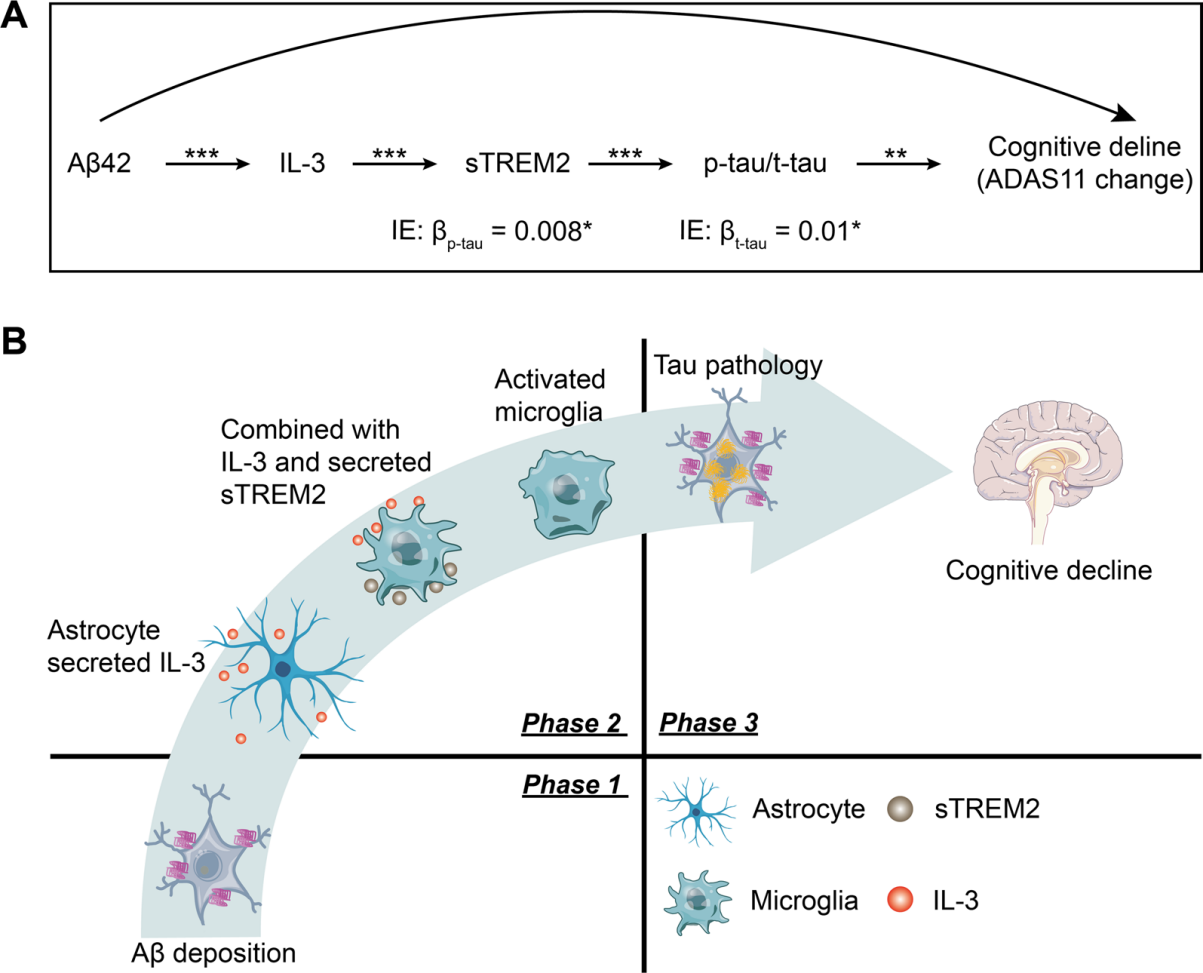
Mediation and schematic representation. **A** The mediation analysis was explored between Aβ42 and ADAS11 annual change: Aβ42 → IL-3 → sTREM2 → p-tau/t-tau → ADAS11 change. *P*-values for mediation effects were calculated by a bootstrap test with 10,000 resampling iterations. *P < 0.05, **P < 0.01 and ***P < 0.001. **B** Schematic representation of the effect of astrocyte–microglia communication on progression of AD pathogenesis. Aβ deposition is the initial step during the development of AD pathogenesis. As the compensative pathway, astrocytes can secrete IL-3 to activate microglia to secrete sTREM2, which leads to microglial activation to clear Aβ deposition. However, when their functions are not powerful enough to remove Aβ deposition, tau-related pathology and neuronal loss will occur, resulting in cognitive decline

Based on the above findings, we tested the synergistic effect of CSF IL-3, CSF sTREM2, and CSF p-tau/t-tau on the association between Aβ42 and cognitive decline: CSF Aβ42 → CSF IL-3 → sTREM2 → CSF p-tau/t-tau → cognitive change. As expected, the significant indirect effect remained (*β*_p-tau_ = 0.008, *p* = 0.03; *β*_t-tau_ = 0.01, *p* = 0.02) (Fig. 5A).

### Sensitivity analysis

To exclude the influence of comorbidities affecting IL-3 on our results, we repeated our primary statistical analysis in the population by excluding those diagnosed with comorbidities affecting IL-3. Details of the participants are presented in Additional file 1: Table S2. As shown in Additional file 1: Tables S3–S5 and Figs. S3, S4, the results of the sensitivity analysis were similar to previous findings.

## Discussion

In the present study, we showed that CSF IL-3 were associated with CSF sTREM2, CSF Aβ42, CSF p-tau, and CSF t-tau as well as subsequent cognitive changes. Our mediation results found that CSF sTREM2 mediates association of CSF IL-3 with p-tau/t-tau, and CSF IL-3 and CSF sTREM2 as key mediators moderated the effect of CSF Aβ42 on CSF p-tau/t-tau. Importantly, the mediation results further demonstrated that both CSF sTREM2 and CSF p-tau/t-tau contributed to the association between CSF IL-3 and cognitive changes. Finally, we further showed that the influence of CSF Aβ42 on cognitive change was affected by the concurrence of CSF IL-3, CSF sTREM2, and CSF p-tau/t-tau. These results provided important human evidence to understand the relationship of cytokine IL-3 with microglial activity and AD pathogenesis as well as cognitive function and supported our hypothesis that glial cell crosstalk, especially astrocyte–microglia communication, may be downstream of amyloid pathology and may play a critical role in the subsequent tau pathology and neurodegeneration as well as cognitive decline in AD (Fig. 5B).

Although astrocytes serve important roles in maintaining the integrity of blood brain barrier (BBB), CNS immune homeostasis, synaptic plasticity, and normal neuronal communication [34–36], the underlying mechanism by which its role implicated in the pathogenesis of AD is poorly understood. There is evidence that increased astrocytic reactivity was around the cortical regions of Aβ accumulation and that astrocytic dysfunction can disrupt the degeneration of Aβ deposition [37, 38]. A recent study demonstrated that CSF IL-3 secreted from astrocytes has the potential to inhibit Aβ deposition [12]. We showed that CSF IL-3 were associated with reduced amyloid pathology, implying that CSF IL-3 may be a key molecule linking the relationship between astrocyte and AD pathology. In addition, our study found that higher baseline CSF IL-3 were associated with reduced cognitive decline. This finding supported the in vivo study suggesting that recombinant (r)IL-3 administration into the cortices of AD mice model improved memory function [12] and the idea that astrocytic protein IL-3 could serve as a novel therapeutic approach for AD.

Consistent with previous studies showing correlations between astrocyte secreted IL-3 and TREM2-signaling [12], we found a positive relationship between CSF IL-3 and CSF sTREM2. Previous studies demonstrated that sTREM2 reflects loss-functional TREM2 and showed that CSF sTREM2 reflects microglial activation and its levels changed dynamically during the pathological course of AD [33, 39–41]. We hypothesized that CSF IL-3 may act in the upstream process of sTREM2 in response to AD pathology, as confirmed by an in vivo study suggesting that IL-3 receptor alpha (IL-3Rα) is TREM2-dependent involved in microglial immune response [12]. Supporting this hypothesis, we found that CSF IL-3 correlated with tau pathology mediated by CSF sTREM2 and that CSF IL-3 and CSF sTREM2 may, at least partially, mediate the influence of Aβ on tau pathology. In addition, we showed that the protective role of CSF IL-3 on cognitive function was influenced by sTREM2 and tau pathology, suggesting that IL-3 may affect cognition via activating the phagocytic abilities of microglia to reduce tau-related pathology. This is supported by previous studies of a distinctive function of IL-3 on the proliferation and programming of microglia and the attenuated effect of IL-3 on tau pathology [13, 20]. These findings bring high clinical value to the role of astrocyte–microglia crosstalk in the progression of AD pathogenesis.

The amyloid cascade hypothesis was believed to be primary evidence for AD progression, suggesting that Aβ accumulation in the brain is an initial step in AD pathogenesis, followed by tau pathology and synaptic loss as well as cognitive decline [42]. This hypothesis has been supplemented by the notion that microglial and astrocytic activation is downstream of Aβ deposition and the upstream of abnormal tau deposition [43]. Indeed, recent studies provide solid evidence that microglial activation increased in response to Aβ plaque metabolism, and its distributions in the brain region were similar to the spread of tau tangles accumulation [41, 44]. Expanding on these studies, we showed, in this study, that CSF IL-3 mediates the association between Aβ and tau pathology, implying that astrocytic activation, similar to microglial activation, has a moderated effect on AD pathology [2]. Furthermore, we found that the concurrence of CSF IL-3, CSF sTREM2, and CSF p-tau/t-tau synergistically mediated the progression from Aβ deposition to the development of cognitive impairment. Our findings support the important concept for AD that after the earlier deposition of Aβ, astrocytes and microglia activate to clear Aβ until their functions are not powerful enough to remove Aβ deposition, and then tau-related pathology and neuronal loss occur, resulting in AD-type cognitive impairment.

Our results may have implications for the ATX(N) system that is based on an extension of the AT(N) system called the Aβ/tau/neurodegeneration to adapt our further knowledge of the pathophysiological mechanisms underlying the AD continuum [45]. Since the ‘X’ component in the ATX(N) system represents candidate biomarkers for additional pathophysiological mechanisms, our results showed that CSF IL-3 and CSF sTREM2 were associated with AD core biomarkers and were involved in the progression from Aβ to tau pathology, and then, they represented astrocyte–microglia communication may be interesting biomarkers for the ‘X’ component. Our findings may also have implications for clinical trials. Since we found results suggesting that CSF IL-3 plays a critical role in the progression of downstream events of Aβ pathology, we may predict that individuals with Aβ deposition without tau pathology would benefit a lot from preventive strategies targeting neuroinflammation. Supporting this notion, recent studies used novel PET tracers to monitor astrocytic activation in the living human brain and they found that astrocytic reactivity was highly selective binding with Aβ deposition and was associated with reduced Aβ deposition in the temporal lobe and sensory areas [46, 47].

The mechanisms linking IL-3 to AD pathology remain elusive. IL-3 has been implicated in multiple biological processes, including neural development, stimulation of cell growth, suppression of apoptosis, and enhancement of hematopoiesis [48]. One possibility of how IL-3 cytokine is linked to AD pathology is its involvement in immune regulation. A mechanism is that IL-3 involves TREM2-dependent signaling to activate microglia for the clearance of Aβ deposition as shown in the mouse model [12]. Another mechanism is that IL-3 against the detrimental effects of Aβ and tau pathology by induced microglial activation through the classic signaling pathway: PI 3-kinase and Jak/STAT pathways [19, 20, 49]. The last possible explanation is that IL-3 is secreted constitutively by a subset of astrocytes [12, 50], which may reflect astrocytic activation that triggers by early Aβ deposition to counteract the detrimental effects of Aβ. However, Aβ pathology may be a double-edged sword for AD progression because excessive Aβ accumulation may cause astrocytic dysfunction and the downstream event of amyloid pathology [46, 51]. These mechanistic explanations remain unclear at this point, and understanding the relationship between IL-3 with AD pathology may prove crucial to designing new therapies to halt the progression of Aβ pathology.

This study has significant strengths. It is the first study using population-based data to systematically the relationship between IL-3 with sTREM2 and AD pathology as well as cognitive function in a well-characterized cohort. Moreover, the possible pathway underlying this process, ranging from the initiated Aβ deposition to cognitive decline, was specified in the current study. Nevertheless, several caveats should be considered when interpreting the current results. First, there are numerous raw values of CSF IL-3 below the least detectable dose (LDD) in the ADNI database, which may cause some results bias. Second, IL-3 data used in this study derived from the Biomarkers Consortium Project that was designed as exploratory and meant for hypothesis and model generation, but not for hypothesis confirmation and model validation. In addition, the use of samples in this study was relatively small. Thus, future work should validate our results using high-sensitivity measurements of CSF IL-3 and large-scale cohorts. Third, the mediation model used in the current observational study gives us a clear explanation of the associations between IL-3 with microglial activation and AD pathology as well as cognitive function, but it cannot allow us to infer causality. Finally, the association between IL-3 and the specific brain regions of AD pathology cannot be detectable in our study due to the limited number of participants with Aβ PET and tau PET in ADNI1 project.

In summary, our results suggested that IL-3 is linked to sTREM2 and mediates the correlation between Aβ pathology to tau pathology. It indicates that IL-3 may be a major factor in the spreading from Aβ pathology to tau pathology to cognitive impairment. These findings have important implications for clinical trials and may encourage future studies on enhancing IL-3 levels as a therapeutic intervention to slow down the development of AD pathology and cognitive decline.

## Supplementary Information

**Additional file 1: Table S1.** Associations of baseline IL-3 and longitudinal cognitive change. **Table S2.** Participant characteristics at baseline by biomarker-defined groups in samples excluding comorbidities. **Table S3.** Associations of baseline IL-3 and CSF biomarkers in samples excluding comorbidities. **Table S4.** Correlations between CSF biomarkers in samples excluding comorbidities. **Table S5.** Associations of baseline IL-3 and longitudinal cognitive change in samples excluding comorbidities. **Figure S1.** Mediation analysis. **Figure S2.** Mediation analysis between IL-3 and cognitive change. **Figure S3.** Mediation analysis in samples excluding comorbidities. **Figure S4.** Mediation in samples excluding comorbidities.

## Data Availability

The dataset supporting the conclusions of this article is available in the ADNI site, http://adni.loni.usc.edu/.

## Abbreviations

Aβ: β-Amyloid
AD: Alzheimer’s disease
ADAS-Cog 11: Alzheimer Disease Assessment Scale-Cognitive Subscale 11
ADAS-Cog 13: Alzheimer Disease Assessment Scale-Cognitive Subscale 13
ADNI: Alzheimer’s Disease Neuroimaging Initiative
ADNI-EF: ADNI Composite Executive Function Score
ADNI-Mem: ADNI Composite Memory Score
ANCOVA: Analysis of covariance
ANOVA: Analysis of variance
CNS: Central neurons system
CSF: Cerebrospinal fluid
FDR: False discovery rate
GFAP: Glial fibrillary acidic protein
IL-3: Interleukin-3
IL-3Rα: IL-3 receptor alpha
MLR: Multiple linear regression
MRM: Multiple reaction monitoring
NFTs: Neurofibrillary tangles
p-tau: Phosphorylated tau
RBM: Rules based medicine
SNAP: Suspected Non-AD Pathology
sTREM2: Soluble triggering receptor expressed on myeloid cells 2
t-tau: Total tau
