# Supplementary material for: Interleukin-3 is associated with sTREM2 and mediates the correlation between amyloid-β and tau pathology in Alzheimer’s disease
Source: J Neuroinflammation. 2022 Dec 29;19:316. doi: 10.1186/s12974-022-02679-5 (PMC9798566; doi:10.1186/s12974-022-02679-5)
Supplement: Supplementary file 1 — Additional file 1: Table S1. Associations of baseline IL-3 and longitudinal cognitive change. Table S2. Participant characteristics at baseline by biomarker-defined groups in samples excluding comorbidities. Table S3. Associations of baseline IL-3 and CSF biomarkers in samples excluding comorbidities. Table S4. Correlations between CSF biomarkers in samples excluding comorbidities. Table S5. Associations of baseline IL-3 and longitudinal cognitive change in samples excluding comorbidities. Figure S1. Mediation analysis. Figure S2. Mediation analysis between IL-3 and cognitive change. Figure S3. Mediation analysis in samples excluding comorbidities. Figure S4. Mediation in samples excluding comorbidities. [file 12974_2022_2679_MOESM1_ESM.docx]

**Interleukin-3 is associated with sTREM2 and mediates the correlation between amyloid-β and tau pathology in Alzheimer’s disease**

Zhi-Bo Wang^1^, Ya-Hui Ma^1^, Yan Sun^1^, Lan Tan^1^, Hui-Fu Wang^1,2,*^, ﻿and Jin-Tai Yu^3,*^, for the Alzheimer’s Disease Neuroimaging Initiative^#^

^1^ Department of Neurology, Qingdao Municipal Hospital, Qingdao University, Qingdao, China.

^2^ Institute of Science and Technology for Brain-Inspired Intelligence, Fudan University, Shanghai, China.

^3^ Department of Neurology and Institute of Neurology, Huashan Hospital, State Key Laboratory of Medical Neurobiology and MOE Frontiers Center for Brain Science, Shanghai Medical College, Fudan University, Shanghai, China.

^*^Corresponding authors

**•Corresponding author’s full address and current Email:**

Hui-Fu Wang, Department of Neurology, Qingdao Municipal Hospital, Qingdao University, No.5 Donghai Middle Road, Qingdao, China; e-mail: wanghuifu2010@126.com.

Jin-Tai Yu, Department of Neurology, Huashan Hospital, Fudan University, No. 12 Wulumuqi Road, Shanghai, China; e-mail: yu-jintai@163.com.

Telephone: 0086-21-52888160

Fax number: 0086-21-62483421

**Table S1: Associations of baseline IL-3 and longitudinal cognitive change.**

|  | Baseline CSF IL-3 × time | | | | |
| --- | --- | --- | --- | --- | --- |
| Cognition | β | Low 95% CI | Up 95% CI | *P* value | N |
| CDR | -0.053 | -0.09 | -0.016 | 0.006 | 310 |
| ADAS11 | -0.069 | -0.105 | -0.033 | <0.001 | 310 |
| ADAS13 | -0.053 | -0.083 | -0.024 | <0.001 | 310 |
| MMSE | 0.059 | 0.018 | 0.1 | 0.006 | 310 |
| ADNI-MEM | 0.028 | 0.011 | 0.045 | 0.001 | 310 |
| ADNI-EF | 0.04 | 0.02 | 0.061 | < 0.001 | 310 |

The relationship between CSF IL-3 and longitudinally assessed cognitive function (CDRSB, ADAS11, ADAS13, MMSE, ADNI-MEM, and ADNI-EF). The normalized regression coefficients (β), 95% confidence interval (CI), and *P* values shown in Table were derived from the interaction term of CSF IL-3 × time in the linear mixed regression model, controlled for CSF IL-3, follow up years, age, sex, education, and *APOE4* status. N indicates the number of participants.

**Table S2: Participant characteristics at baseline by biomarker-defined groups in samples excluding comorbidities.**

| Characteristic | Stage 0 | Stage 1 | Stage 2 | SNAP | *P* value | |
| --- | --- | --- | --- | --- | --- | --- |
| N | 41 | 28 | 98 | 29 | - | |
| Age, mean (SD), y | 75.01 (5.23) | 74.99 (5.47) | 75.01 (7.10) | 78.02 (6.14) | 0.142 | |
| Female, n (%) | 14 (34.1) | 6 (21.4) | 43 (43.9) | 9 (31.0) | 0.139 | |
| Education, mean (SD), y | 15.54 (3.03) | 15.75 (3.73) | 15.69 (3.15) | 15.55 (2.81) | 0.988 | |
| APOE ε4 carriers, n (%) | 4 (9.8) | 14 (50.0) | 72 (73.5) | 5 (17.2) | <0.001 | |
| MMSE score, mean (SD) | 28.59 (1.30) | 26.21 (2.42) | 25.96 (2.70) | 27.97 (1.95) | <0.001 | |
| AD diagnosis, n (%) | 1 (2.4) | 5 (17.9) | 32 (32.7) | 4 (13.8) | <0.001 | |
| CSF IL-3, mean (SD) | -2.12 (0.34) | -2.42 (0.30) | -2.20 (0.30) | -1.93 (0.28) | <0.001 | |
| CSF biomarkers, mean (SD), pg/ml | | | | | |  |
| CSF sTREM2 | 4506.33 (2150.52) | 3014.51 (1320.98) | 4599.74 (2075.24) | 5805.51 (2015.21) | <0.001 | |
| CSF Aβ42 | 1450.90 (251.44) | 616.18 (184.87) | 620.45 (174.18) | 1605.11 (502.36) | <0.001 | |
| CSF p-tau | 16.94 (2.64) | 16.62 (3.84) | 36.39 (10.35) | 29.78 (10.65) | <0.001 | |
| CSF t-tau | 193.12 (28.60) | 179.66 (36.18) | 360.70 (93.69) | 324.27 (86.34) | <0.001 | |

Abbreviations: Aβ, β-amyloid; AD, Alzheimer’s disease; CSF, cerebrospinal fluid; IL-3, interleukin-3; MMSE, Mini-Mental State Examination; p-tau, phosphorylated tau; t-tau, total tau.

﻿*P* values were computed with the one-way analysis of covariance test for age, education, MMSE score, CSF IL-3, CSF sTREM2, CSF Aβ42, CSF t-tau, CSF p-tau; with the χ^2^ test for sex and *APOE* status.

**Table S3: Associations of baseline IL-3 and CSF biomarkers in samples excluding comorbidities.**

|  | CSF IL-3 | | | | |
| --- | --- | --- | --- | --- | --- |
| CSF biomarkers | β | Low 95% CI | Up 95% CI | *P* value | N |
| sTREM2 | 0.483 | 0.358 | 0.608 | <0.001 | 204 |
| Aβ42 | 0.358 | 0.243 | 0.473 | <0.001 | 250 |
| t-tau | 0.237 | 0.118 | 0.356 | <0.001 | 250 |
| p-tau | 0.182 | 0.061 | 0.303 | 0.003 | 250 |

Associations of CSF IL-3 with CSF sTREM2, CSF Aβ42, CSF p-tau, and CSF t-tau in samples excluding comorbidities. The normalized regression coefficients (β), 95% confidence intervals, and P values s were derived from multiple linear regression, adjusting age, sex, education, and APOE4 status.

**Table S4: Correlations between CSF biomarkers in samples excluding comorbidities.**

|  | IL-3 | | sTREM2 | | Aβ42 | | p-tau | | t-tau | |
| --- | --- | --- | --- | --- | --- | --- | --- | --- | --- | --- |
|  | r | *P* value | r | *P* value | r | *P* value | r | *P* value | r | *P* value |
| IL-3 | - | - | 0.51 | <0.001 | 0.32 | <0.001 | 0.27 | <0.001 | 0.33 | <0.001 |
| sTREM2 | 0.51 | <0.001 | - | - | 0.25 | <0.001 | 0.35 | <0.001 | 0.39 | <0.001 |
| Aβ42 | 0.32 | <0.001 | 0.25 | <0.001 | - | - | -0.15 | 0.04 | -0.07 | 0.32 |
| p-tau | 0.27 | <0.001 | 0.35 | <0.001 | -0.15 | 0.04 | - | - | 0.98 | <0.001 |
| t-tau | 0.33 | <0.001 | 0.39 | <0.001 | -0.07 | 0.32 | 0.98 | <0.001 | - | - |

Associations of CSF IL-3 with CSF sTREM2, CSF Aβ42, CSF p-tau, and CSF t-tau in samples excluding comorbidities. The coefficients (r) and P value are derived from Spearman partial correlation after controlled age, sex, education, and *APOE4* status.

**Table S5: Associations of baseline IL-3 and longitudinal cognitive change in samples excluding comorbidities.**

|  | Baseline CSF IL-3 × time | | | | |
| --- | --- | --- | --- | --- | --- |
| Cognition | β | Low 95% CI | Up 95% CI | *P* value | N |
| CDR | -0.043 | -0.084 | -0.003 | 0.036 | 266 |
| ADAS11 | -0.06 | -0.098 | -0.023 | 0.002 | 266 |
| ADAS13 | -0.048 | -0.078 | -0.018 | 0.002 | 266 |
| MMSE | 0.041 | -0.002 | 0.084 | 0.064 | 266 |
| ADNI-MEM | 0.025 | 0.008 | 0.043 | 0.006 | 266 |
| ADNI-EF | 0.038 | 0.015 | 0.061 | 0.001 | 266 |

The relationship between CSF IL-3 and longitudinally assessed cognitive function (CDRSB, ADAS11, ADAS13, MMSE, ADNI-MEM, and ADNI-EF) in samples excluding comorbidities. The normalized regression coefficients (β), 95% confidence interval (CI), and *P* values shown in Table were derived from the interaction term of CSF IL-3 × time in the linear mixed regression model, controlled for CSF IL-3, follow up years, age, sex, education, and *APOE4* status. N indicates the number of participants.

**
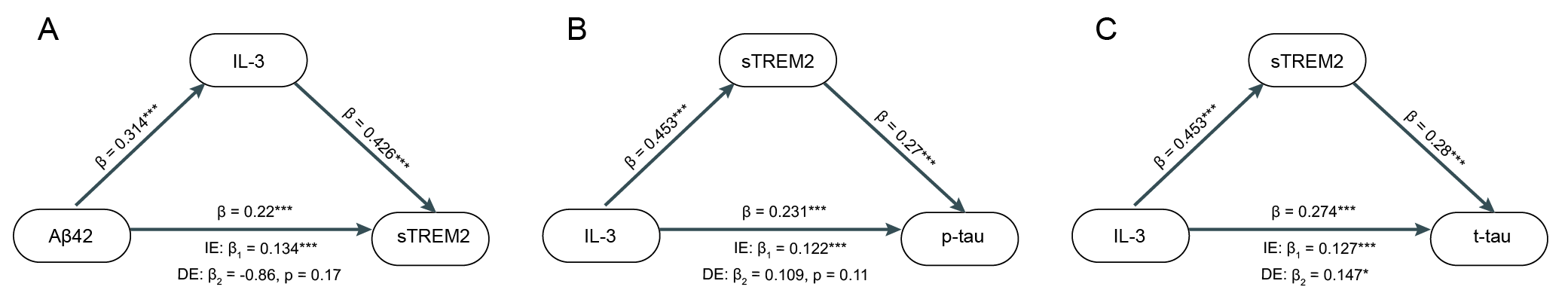
Figure S1. Mediation analysis.**

﻿Three mediation pathways were conducted between Aβ42 and p-tau/t-tau: **A** Aβ42 → IL-3 → sTREM2; **B** IL-3 → sTREM2 → p-tau; **C** IL-3 → sTREM2 → t-tau. All mediation paths are adjusted by covariates (age, sex, education, and *APOE4* status). *P*-values for mediation effects were calculated by a bootstrap test with 10,000 resampling iterations. **P* < 0.05, ***P* < 0.01 and ****P* < 0.001. *APOE*, *apolipoprotein E*; CSF, cerebrospinal fluid; IL-3, interleukin-3; sTREM2, soluble triggering receptor expressed on myeloid cells.


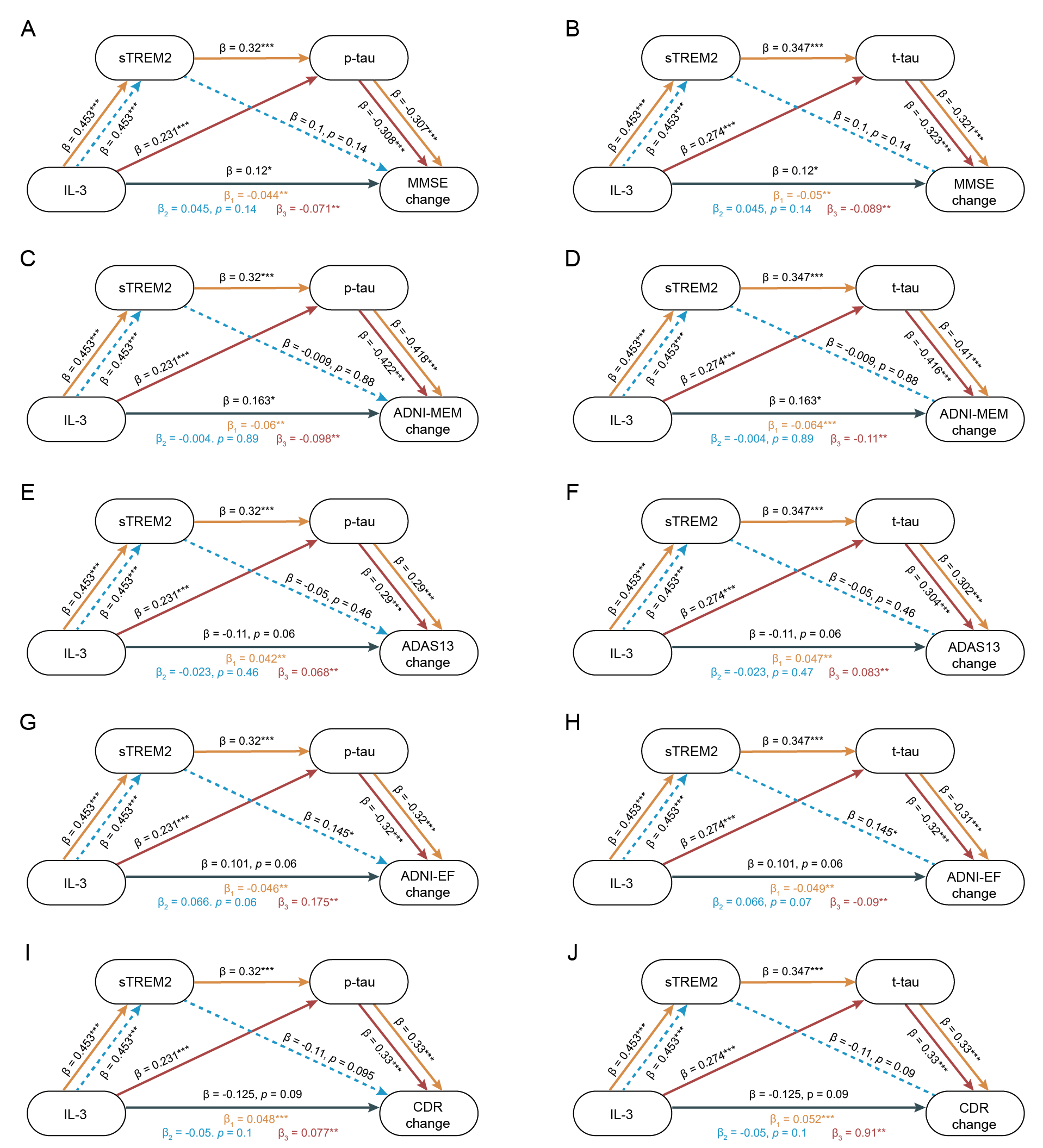


**Figure S2. Mediation analysis between IL-3 and cognitive change.**

**A-B** Three mediation pathways were conducted between IL-3 and MMSE annual change: 1) IL-3 → sTREM2 → p-tau/t-tau → MMSE change; 2) IL-3 → sTREM2 → MMSE change; 3) IL-3 → p-tau/t-tau → MMSE change. **C-D** Three mediation pathways were assessed between IL-3 and ADNI-MEM annual change: 1) IL-3 → sTREM2 → p-tau/t-tau → ADNI-MEM change; 2) IL-3 → sTREM2 → ADNI-MEM change; 3) IL-3 → p-tau/t-tau → ADNI-MEM change. **E-F** Three mediation pathways were assessed between IL-3 and ADAS13 annual change: 1) IL-3 → sTREM2 → p-tau/t-tau → ADAS13 change; 2) IL-3 → sTREM2 → ADAS13 change; 3) IL-3 → p-tau/t-tau → ADAS13 change. **G-H** Three mediation pathways were assessed between IL-3 and ADNI-EF annual change: 1) IL-3 → sTREM2 → p-tau/t-tau → ADNI-EF change; 2) IL-3 → sTREM2 → ADNI-EF change; 3) IL-3 → p-tau/t-tau → ADNI-EF change. **I-J** Three mediation pathways were assessed between IL-3 and CDR annual change: 1) IL-3 → sTREM2 → p-tau/t-tau → CDR change; 2) IL-3 → sTREM2 → CDR change; 3) IL-3 → p-tau/t-tau → CDR change. These three pathways are presented using yellow, blue, and red lines. All mediation paths are adjusted by covariates (age, sex, education, and *APOE4* status). *P*-values for mediation effects were calculated by a bootstrap test with 10,000 resampling iterations. The dotted line indicates that the indirect effect is not significant (*P* ≥ 0.05), and the solid line indicates that the indirect effect is significant (*P* < 0.05). **P* < 0.05, ***P* < 0.01 and ****P* < 0.001. ADAS 13: Alzheimer Disease Assessment Scale 13; ADNI-EF, ADNI composite executive function score; ADNI-MEM, ADNI composite memory score; *APOE*, *apolipoprotein E*; CDR, Clinical Dementia Score; CSF, cerebrospinal fluid; IL-3, interleukin-3; MMSE, Mini-Mental State Exam; sTREM2, soluble triggering receptor expressed on myeloid cells.

**
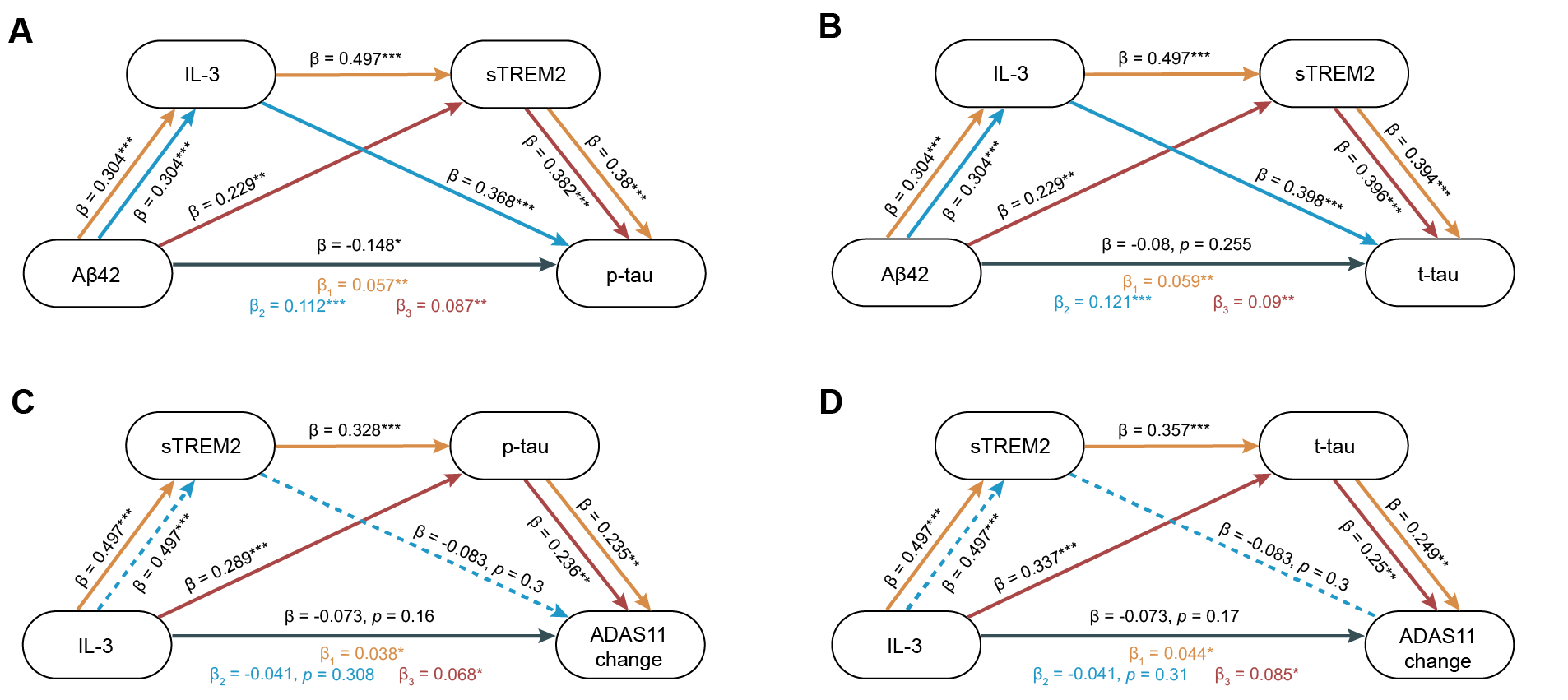
**

**Figure S3. Mediation analysis in samples excluding comorbidities.**

(**A-B**) Three mediation pathways were conducted between Aβ42 and p-tau/t-tau: 1) Aβ42 → IL-3 → sTREM2 → p-tau/t-tau; 2) Aβ42 → IL-3 → p-tau/t-tau; 3) Aβ42 → sTREM2 → p-tau/t-tau. (**C-D**) Three mediation pathways were assessed between IL-3 and ADAS11 annual change: 1) IL-3 → sTREM2 → p-tau/t-tau → ADAS11 change; 2) IL-3 → sTREM2 → ADAS11 change; 3) IL-3 → p-tau/t-tau → ADAS11 change. These three pathways are presented using yellow, blue, and red lines. All mediation paths are adjusted by covariates (age, sex, education, and *APOE4* status). The β coefficients in each path and *P*-values for mediation effects were calculated by a bootstrap test with 10,000 resampling iterations. The dotted line indicates that the indirect effect is not significant (*P* ≥ 0.05), and the solid line indicates that the indirect effect is significant (*P* < 0.05). **P* < 0.05, ***P* < 0.01 and ****P* < 0.001.


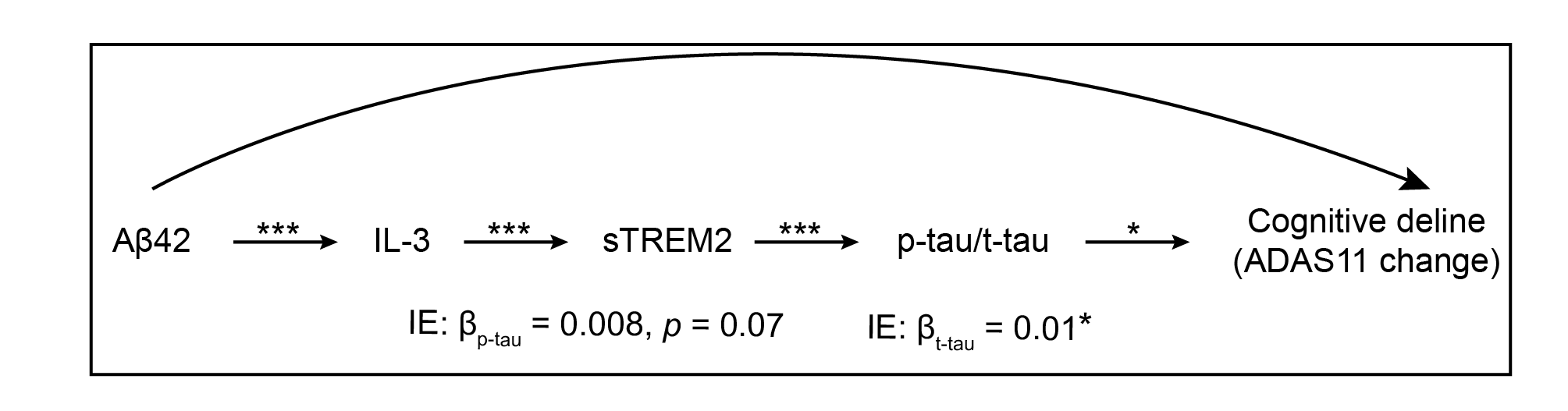


**Figure S4. Mediation in samples excluding comorbidities.**

The mediation analysis was explored between Aβ42 and ADAS11 annual change: Aβ42 → IL-3 → sTREM2 → p-tau/t-tau → ADAS11 change. *P*-values for mediation effects were calculated by a bootstrap test with 10,000 resampling iterations. **P* < 0.05, ***P* < 0.01 and ****P* < 0.001.
